# Editorial: Molecular targeting of the tumor microenvironment for therapeutics in cancer metastasis

**DOI:** 10.3389/fmolb.2023.1178488

**Published:** 2023-03-21

**Authors:** Subramanyam Dasari, Prabhakar Pitta Venkata, Uday P. Pratap, Ram Mohan Ram Kumar

**Affiliations:** ^1^ School of Medicine, Indiana University Bloomington, Bloomington, IN, United States; ^2^ The University of Texas Health Science Center at San Antonio, San Antonio, TX, United States; ^3^ Rajiv Gandhi Centre for Biotechnology (RGCB), Thiruvananthapuram, Kerala, India

**Keywords:** tumor microenvironment (TME), metastasis, extracellular matrix (ECM), immune checkpoints, legumain (LGMN)

More than 90% of cancer mortality is due to metastasis; however, the essential mechanisms driving this multistep process, from the primary site to the development of metastatic cells at the secondary sites, remain tenuous. The communication between the cancer cells and the associated stromal cells starts at the stages of tumor formation and continues during growth, invasion, intravasation, and establishment at the secondary site called metastasis. This cross-talk between the cancer cells and stromal cells establishes a tumor microenvironment (TME). TME dynamically regulates cancer progression, promotes immune evasion, and influences the therapeutic outcome. Hence, strategies to therapeutically target the TME have emerged as a promising approach for standard-of-care therapies. The TME comprises the extracellular matrix (ECM); secreted molecules (growth factors, cytokines, chemokines, and extracellular vesicles); stromal cells such as cancer-associated fibroblasts (CAF), pericytes, endothelial cells, adipose cells, and mesenchymal cells; immune cells, including T and B lymphocytes; tumor-associated macrophages (TAM); dendritic cells (DC); natural killer (NK) cells; neutrophils; and the blood and lymphatic vascular networks, which are collectively enmeshed and in communication with each other and with the heterogeneous cancer cells themselves. Because of this complexity, various strategies have been developed to therapeutically target the TME. Despite the excellent advances that were made in the last decade, some improvements are still required at each stage of TME development and metastasis.

The main aim of this Research Topic is to unveil novel therapeutic strategies to target the TME and thereby regulate metastasis. The themes covered by this Research Topic include the identification of the novel tumor microenvironment-related prognostic signature (Li et al.), how tumor microenvironment components regulate the resistance to gemcitabine and the immune checkpoint inhibitor in pancreatic ductal adenocarcinoma (PDAC) (Hsu et al.), how the stromal CCN family protein contributes to the poor prognosis in lower grade glioma by modulating immunity, matrix, stemness, and metabolism (Liu et al.), and the role of legumain (LGMN) in tumor development and its progression associated with the tumor microenvironment (Khan et al.).

Li et al. have investigated a TME-based prognostic model to predict the efficacy of immunotherapy in HCC patients. They studied the transcriptomic data of 374 HCC patients from the TCGA database, based on the TME scores (immune/stromal/estimate scores) and tumor purity. Gene set enrichment analyses (GSEA) were performed to investigate the underlying biological functions and pathways of this risk signature. Additionally, the research team also evaluated the possible correlation of the risk signature with TME immune cell infiltration, immune checkpoint inhibitor (ICI) treatment response, single-nucleotide polymorphisms (SNPs), and drug sensitivity. They identified 5 genes TME risk signatures (DAB2, IL18RAP, RAMP3, FCER1G, and LHFPL2) to predict the prognosis of patients with HCC. Patients with a low-risk score were found to have higher levels of tumor-infiltrating immune cells and higher expression of immune checkpoints, which may be more sensitive to immunotherapy.

Hsu et al. have explored and characterized an extremely immunosuppressive tumor microenvironment (TME) in pancreatic ductal adenocarcinoma (PDAC). Chemoresistance (gemcitabine—GEM) can be conferred by the components through modulating drug metabolism and enhancing antiapoptotic effects, and similarly, gemcitabine-activated CAFs induce accumulation of ECM by forming a physical barrier and considerably compromising the access of GEM to tumor compartments. To overcome these issues, Hsu et al. found the chip-based exosome analysis could predict treatment response and prognosis due to the abundance of TME-derived exosomes detected in body fluids (e.g., blood and urine) and that their cargos are indicative of PDAC progression. The team also proposed precision medicine, in which single-cell sequencing of TME components and immune profiling provide information to establish personalized treatments. Subsequently, drug candidates could be encapsulated in exosomes to improve drug penetration. This review provides new insights into clinical applications, including personalized medicine, disease monitoring, and drug carriers.

Liu et al. have investigated CCN family stromal proteins (CCN4) to regulate immunity, matrix, stemness, and metabolism in lower-grade glioma (LGG). Based on the RNA-seq profiles from multiple cohort studies, CCN4 was associated with poor prognosis in LGG. In addition, they explored the functions of CCNs in LGG and found that CCNs were closely associated with gene mutations (EGFR, PTEN, and NF1), high inflammation, ECM, stemness and metabolic abnormalities, and immune escape in LGG. These findings outlined the role of the CCN family in LGG and provided a potential and promising therapeutic target in LGG.

Khan et al. have reported that legumain (LGMN) is a member of the C13 family of a cysteine protease enzyme called an asparaginyl endopeptidase (AEP). Elevated expression of LGMN is reported in several cancers including breast, prostate, and liver cancers and in the macrophages of the tumor microenvironment. Hence, LGMN is considered a key protein involved in the regulation of tumor angiogenesis, invasion, and metastasis. Recent studies have reported that targeting LGMN using siRNA or small molecular inhibitors reduces cancer cell growth *in vitro* and shrinks tumor size *in vivo*. Furthermore, legumain can also be used as a diagnostic marker due to its expression being significantly lower in normal cells compared to tumors or tumor-associated macrophages (TAMs). Since LGMN plays an important role in the immune system and is preferentially over-expressed in tumor microenvironments and tumor tissues, targeting LGMN may be a promising strategy for tumor immunotherapy.

